# Medical and legal point of view for low-vision patients

**Published:** 2018

**Authors:** Camelia-Margareta Bogdănici, Ştefan Tudor Bogdănici, Diana Elena Săndulache, Carmen-Mariana Diaconu

**Affiliations:** * ”Grigore T. Popa” University of Medicine and Pharmacy, Iaşi, Romania; Surgery II Department, Discipline of Ophthalmology, “Sf. Spiridon” Hospital, Iași, Romania; ** Preventive Department and Interdisciplinarity Department, Discipline of Public Health and Sanitary Management, ”Grigore T. Popa” University of Medicine and Pharmacy, Iași, Romania; *** “Ion Ionescu de la Brad” University of Agricultural Sciences and Veterinary Medicine, Agriculture Faculty, Iași, Romania

**Keywords:** low-vision, ocular parameters, medical legislation, visual impairment

## Abstract

The aim of the study was to highlight the medical and legal difficulties in framing low-vision patients for certification. We performed a retrospective observational study conducted from January 2013 to January 2016, on 63 patients with the mean age of 16.37±3.34 years, evaluated at the Ophthalmology Clinic from “Sf. Spiridon” Hospital, Iași, in order to release a medical certificate required at the Expertise Board. The clinical parameters observed were visual acuity (VA) with correction, objective refraction (in Spherical Equivalent - SEq), intraocular pressure, slit lamp examination of the anterior pole, fundus examination, orthoptic eye exam, and ocular ultrasonography (in selected cases). The main causes for the decreased visual acuity found are *refractive or strabic amblyopia* determined by: high myopia (28.57%), esotropia (19.04%), astigmatism (17.46); *congenital diseases* - congenital nystagmus (12.69%), congenital cataract (7.93%), microphthalmia (7.93%); *acquired diseases* - retinopathy of prematurity (9.52%), optic nerve atrophy (7.93%), bandelette keratopathy (6.34); *ocular trauma* (7.93%). In 52.38% of the cases for the RE and 53.96% of the cases for the LE, decreased visual acuity was caused by an irreversible condition and could not be improved. Patients come every year for reevaluation in order to receive the medical certificate required at the Expertise Board. Evaluating the patient for a certificate for visual impairment is a time consuming process due to the high number of investigations necessary and, sometimes, difficult collaboration with the patient with associated general pathology. It also requires knowledge of frequently changing legislation to complete legal forms for patients with visual impairment. A medical certificate may now be issued with a validity of up to four years, given that certain diseases are irreversible and visual functional status does not change over time.

## Introduction

Visual impairment is defined as a functional limitation of the eye(s) or visual system. Symptoms felt by the patient are reduced visual acuity or contrast sensitivity, visual field loss, diplopia, photophobia, visual perceptual difficulties or visual distortion [**1**].

There are 285 million people estimated to be visually impaired worldwide. Of these, 39 million are blind and 246 have low vision [**2**]. It is estimated that there are 19 million children of less than 15 years worldwide who are visually impaired, and that 12 million have a refractive error, which can be easily corrected and 1.4 million are irreversibly blind for the rest of their lives [**2**]. 

Patients with visual impairment are divided into two categories: individuals with low vision and individuals with blindness with distinct characteristics [**3**] (**Table 1**). Students with low vision have residual vision and they are able to use special equipment to read printed materials. Blindness is characterized by a total loss of vision and conditions in which individuals need to rely predominantly on vision substitution skills, meaning that a blind student does not use vision in the learning process [**4**].

**Table 1:** Classification of visual impairment (after World Health Organization, 2016)

Category	Presenting distance visual acuity	
	Worse than:	Equal to or better than:
0 Mild or no visual impairment		6/18
		3/10 (0.3)
		20/70
1 Moderate visual impairment	6/18	6/60
	3/10 (0.3)	1/10 (0.1)
	20/70	20/200
2 Severe visual impairment	6/60	3/60
	1/10 (0.1)	1/20 (0.05)
	20/200	20/400
3 Blindness	3/60	1/60*
	1/20 (0.05)	1/50 (0.02)
	20/400	5/300 (20/1200)
4 Blindness	1/60*	Light perception
	1/50 (0.02)	5/300 (20/1200)
5 Blindness	No Light perception	
9	Undetermined or unspecified	
	*or counts fingers (CF) at 1 metre	

Visual impairment causes unfavorable consequences for both the individual and the collectivity. Blindness generates psychological, social, and economic difficulties and it can lead to loss of autonomy and self-esteem [**3**].

## Material and methods

The study was a retrospective observational one conducted from January 2013 to January 2016, on 63 patients with a mean age of 16.37±3.34 years (limits 13 - 27 years), evaluated at Ophthalmology Clinic from “Sf. Spiridon” Hospital, Iasi, in order to release a medical certificate required at the Expertise Board. In the selected cases, the majority of patients (68%) were male. 63.76% were from rural areas, which highlighted the late addressability of patients for a specialized examination. The clinical parameters observed were:

- visual acuity (VA) with correction (with Snellen chart),

- objective refraction (OR) (Topcon autorefractometer),

- intraocular pressure (Goldmann aplanotonometer), 

- slit lamp examination of the anterior pole,

- fundus examination (direct ophthalmoscopy), 

- orthoptic eye exam (synoptophore and prims),

- ocular ultrasonography (in selected cases). 

Refraction values were calculated using the spherical equivalent (SEq), which represents the sphere’s value plus half of the cylinder’s value.

The study showed the difficulties for an ophthalmologist to known the changing of legal parameters and laws to complete all the papers for the Expertise Board. These parameters change very often and make difficulties for criteria for visual handicap. It is necessary to be in a permanent connection with a lawyer to complete all the papers for the Expertise Board in good condition.

## Results

**Fig. 1:**
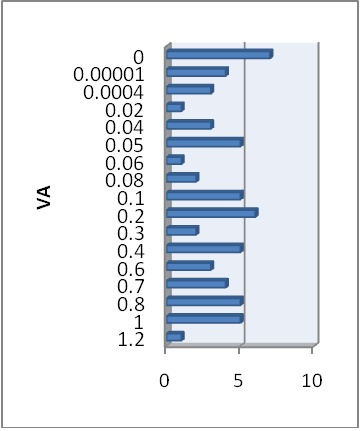
VA – distribution RE

**Fig. 2:**
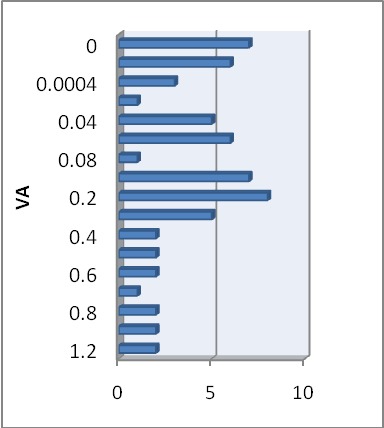
VA – distribution LE

Mean visual acuity on Snellen chart (with correction) for the right eye (RE) was 0.32±0.35 (limits between 0 and 1.2) (**Fig. 1**). For the left eye (LE), the mean visual acuity with correction was 0.23±0.31, with the same limits as the RE (**Fig. 2**). For the RE (after World Health Organization, 2003), 17.7% of the cases had moderate visual impairment, 14.51% had severe visual impairment and 27.41% fit into a blindness category. For the LE, 24.19% of the cases had moderate visual impairment, 11.29% had severe visual impairment, and 35.47% fit into a blindness category. Bilateral visual impairment was present in 51.61% of the cases; unilateral visual impairment in 27.41% and 20.9% had mild or no visual impairment.

**Fig. 3:**
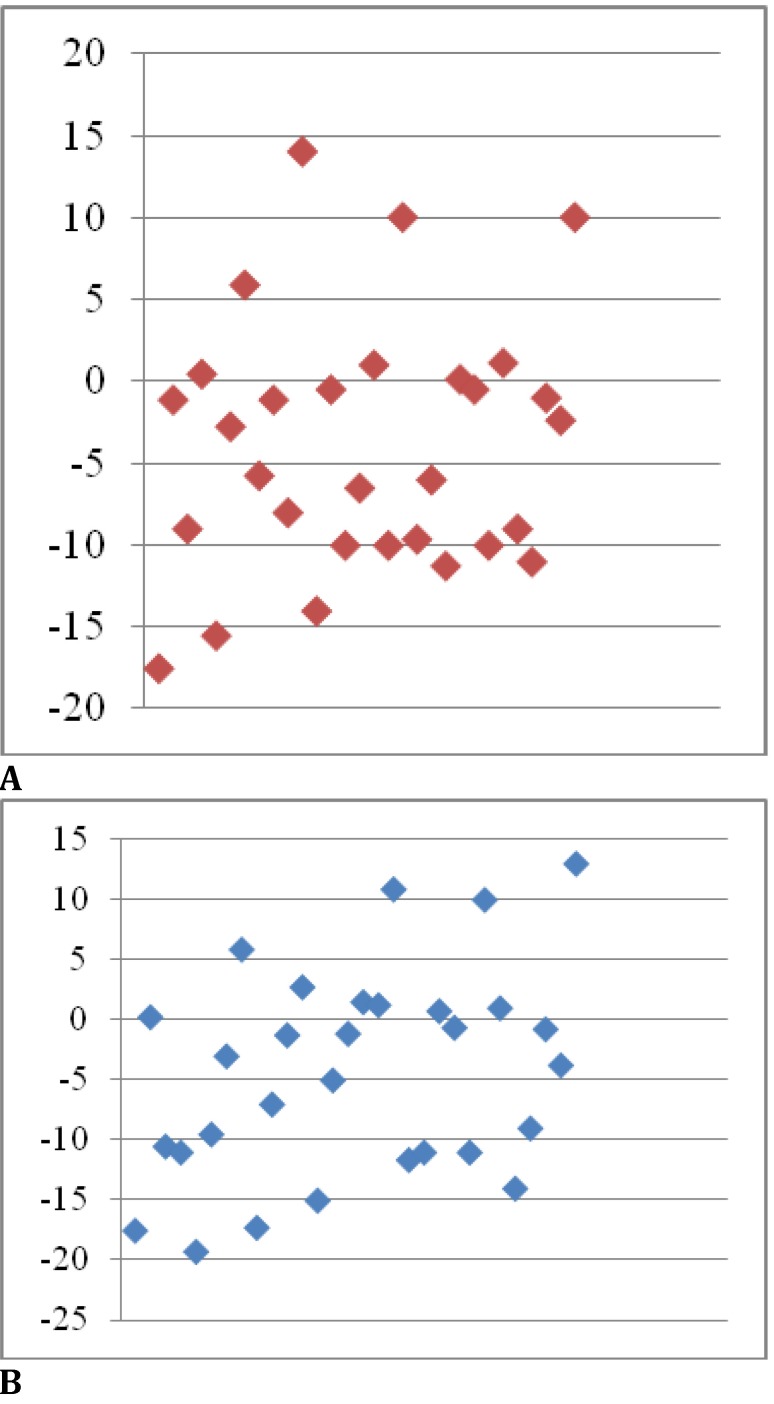
Refractive errors: a - refractive errors for RE and b - refractive errors for LE

Regarding the optical correction, it was necessary in 50% of the cases for the RE and in 50% of the cases for the LE. The refractive errors (in SEq) for the RE had a mean value of 4±7.64 D (limits between -17.5 D and +14 D) (**Fig. 3.a**) and for the LE 4.4±8.57 D (limits between -19.25 D and +13) (**Fig. 3.b**).

Of the 63 patients, 44.4% had changes in fundus examination for the RE and 47.61% for the LE. Changes in the anterior pole were found in 28.5% of the cases for the RE and in 28.5% of the cases for the LE. For 10 patients (15.87%), eye ultrasound (Mode A and B) was required, 9.2% showed changes in the RE and 7.93% in the LE.

Certification depending on the value of visual acuity (by Order no. 707/538/2014) was the following: *for the RE* - 8% were slightly sight impaired, 18% medium sight impaired and 24% severely sight impaired; *for the LE* - 11.29% were slightly sight impaired, 19.35% medium sight impaired and 27.42% severely sight impaired. In 52.38% of the cases for the RE and 53.96% of the cases for the LE, decreased visual acuity was caused by an irreversible condition and could not be improved. 

**Fig. 4:**
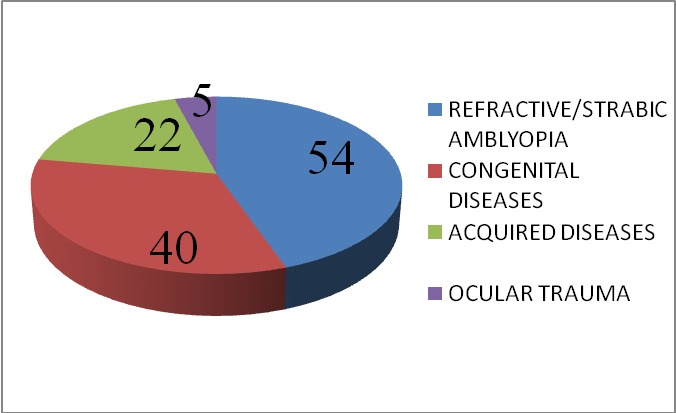
Causes of decreased visual acuity

The main causes for the decreased visual acuity found were (**Fig. 4**): *refractive or strabic amblyopia* - high myopia (28.57%), esotropia (19.04%), astigmatism (17.46%), myopic anisometropia (6.34%), high hyperopia (4.76%), hyperopic anisometropia (1.58%); *congenital diseases* - congenital nystagmus (12.69%), congenital cataract (7.93%), microphthalmia (7.93%), persistent of primitive vitreous (6.34%), Stargardt disease (6.34%), pigmentary retinopathy (3.17%), congenital glaucoma (3.17%); *acquired diseases* - retinopathy of prematurity (9.52%), optic nerve atrophy (7.93%), bandelette keratopathy (6.34%), ocular toxoplasmosis (4.76%), secondary glaucoma (3.17%); *ocular trauma* (7.93%). Most patients had multiple causes for decreased visual acuity.

Patients come every year for reevaluation in order to receive the medical certificate required at the Expertise Board. After certification depending on the value of visual acuity Order no. 707/538/2014, 27,42% of the patients had severe sight impaired, 18% medium sight impaired and 8% slightly sight impaired (**Table 2**).

**Table 2:** Classification of visual impaired

Vision	RE	LE
slightly sight impaired	8%	11.9%
medium sight impaired	18%	19.35%
severely sight impaired	24%	27.42%

## Discussions

Visual impairment is defined as blindness or low vision [**5**] and is associated with an important limitation in ocular function. Psychological distress, difficulties in activities of daily living and low health-related quality of life has been reported in patients with visual impairment [**6**,**7**]. Amblyopia, from the Greek *amblus* (dull) and *ops* (eye), is a common cause of vision loss affecting between 1% and 3% of the population. It has been reported to be more common in left eyes than right eyes [**8**] and is, according to the National Eye Institute, the leading cause of unilateral vision loss in the under-70 population. Amblyopia affects both near and distance visual acuity equally [**9**]. The early detection and treatment improve multiple quality of life indicators and treatments are very cost-effective in terms of benefits derived compared with costs of care [**10**,**11**]. 

According to the data for 2010, 80% of the visual impairment including blindness is avoidable. The two main causes of visual impairment in the world are uncorrected refractive errors (42%) and cataract (33%) [**2**]. The leading cause of unilateral vision loss in under-70 years old population [**10**]. It has been reported to be more common in left eyes than in the right eyes [**8**].

The prevalence of childhood blindness varies according to socioeconomic status [**12**]. In very low income countries with high mortality rates in children younger than 5 years, the prevalence may be as high as 1.5 per 1000 children, in contrast with high-income countries where the prevalence is five times less [**13**]. Strabismus or strabismus surgery history is present in 37,5% of the children with amblyopia, anisometropia in 34,4%, both conditions in 18,8% of the cases [**14**]. The prevalence of visual impairment in European populations shows a definite increasing trend from north to South [**15**].

Visually impaired persons experience mental health problems related to their vision loss and they might need and want help for this [**16**]. Vision loss is significantly associated with depression and certain traits of personality (specifically neuroticism and conscientiousness), independent of the severity of vision loss, and duration of vision loss [**17**].

In Romania, the certificates for visual impairment are given according to three National Orders: 762/ 1992 from August 2007, 707/ 538 from May 2014 and 874/ 554/ 2016 from April 2016. Given the large number of normative acts that also include the orders of the Ministry of Health, public policies generate the implementation of problems in the absence of training for medical professionals. It is necessary to create a center coordinated by the Public Health Department, subordinated to the Ministry of Health. This center would inform medical professionals about the specific documents and forms required for the issuance of a medical certificate to patients with a degree of disability, assessed according to the legislation on the matter. Also, this center would help both in the continuous professional training of medical staff and in the benefit of patients, who would receive a complete file for the specific assessment required for the issuance of the certificate attesting a certain degree of disability. 

## Conclusions

Evaluating the patient for certification is a time consuming process due to the high amount of investigations necessary and, sometimes, difficult collaboration with the patient with an associated general pathology. It also requires knowledge of frequently changing legislation. A medical certificate may now be issued with a validity of up to four years, given that certain diseases are irreversible and visual functional status does not change over time. From a legislative point of view, it is necessary for patients already confirmed with a degree of disability, who attest the impossibility of acquiring work capacities in relation to the already performed medical tests, not to re-take those specific tests, unless the received medical treatment could have restored one’s work capacity.

**Conflict of interest**

The authors do not have a financial interest/arrangement or affiliation with one or more organizations that could be perceived as a real or apparent conflict of interest in the context of the subject of the manuscript. 
